# 6-Valent Virus-Like Particle-Based Vaccine Induced Potent and Sustained Immunity Against Noroviruses in Mice

**DOI:** 10.3389/fimmu.2022.906275

**Published:** 2022-05-23

**Authors:** Wenli Hou, Lihui Lv, Yihan Wang, Man Xing, Yingying Guo, Di Xie, Xin Wei, Xiuyue Zhang, Hui Liu, Jiling Ren, Dongming Zhou

**Affiliations:** ^1^ Key Laboratory of Bio resource and Eco-environment, College of Life Science, Sichuan University, Chengdu, China; ^2^ Department of Pathogen Biology, School of Basic Medical Sciences, Tianjin Medical University, Tianjin, China; ^3^ R&D Centre, Chengdu Kanghua Biological Products Co., Ltd, Chengdu, China

**Keywords:** 6-valent norovirus vaccines, virus-like particles (VLPs), dose-dependent tendency, humoral immune responses, T-cell responses

## Abstract

Norovirus is a major cause of acute gastroenteritis worldwide, and no vaccine is currently available. The genetic and antigenic diversity of Norovirus presents challenges for providing broad immune protection, which calls for a multivalent vaccine application. In this study, we investigated the possibility of developing a virus-like particle (VLP)-based 6-valent Norovirus vaccine candidate (Hexa-VLPs) that covers GI.1, GII.2, GII.3, GII.4, GII.6, and GII.17 genotypes. Hexa-VLPs (30 µg) adjuvanted with 500 µg of aluminum hydroxide (alum) were selected as the optimal immunization dose after a dose-escalation study. Potent and long-lasting blockade antibody responses were induced by 2-or 3-shot Hexa-VLPs, especially for the emerging GII.P16-GII.2 and GII.17 (Kawasaki 2014) genotypes. Hexa-VLPs plus alum elicited Th1/Th2 mixed yet Th2-skewed immune responses, characterized by an IgG1-biased subclass profile and significant IL-4^+^ T-cell activation. Notably, simultaneous immunization with a mixture of six VLPs revealed no immunological interference among the component antigens. These results demonstrate that Hexa-VLPs are promising broad-spectrum vaccines to provide immunoprotection against major GI/GII epidemic strains in the future.

## Introduction

Norovirus (NoV) is a major cause of acute gastroenteritis (AGE), causing 685 million infections and 210,000 deaths worldwide each year (1). NoV is a non-enveloped, single-stranded, positive-sense RNA virus, which can be classified into 10 genogroups and over 48 genotypes according to the similarity of RNA-dependent RNA polymerase (RdRp-NS7) or major capsid protein VP1 (2, 3).

Humans are mainly infected by GI, GII, and GIV genogroups (4). However, the NoV burden is dynamic because circulating NoV strains vary annually. The GII.4 genotype, which is recognized as the major cause of large epidemics globally, has undergone epochal evolution with new GII.4 variants emerging every 2-4 years and replacing the previously dominant strains (5). A meta-analysis studied on all AGE caused by NoVs from mainland China before 2017 indicated GII.4 was the dominant strain followed by GII.3, GII.17 (6). During the winter of 2014-2015, an emergent variant of the GII.17 genotype, Kawasaki 2014, predominated in Hong Kong and outcompeted GII.4 Sydney 2012 in hospitalized cases (7). In 2016, a recombinant norovirus strain, GII.P16-GII.2, led to 79% of the 56 outbreaks, indicating that this strain became a dominant strain in China (8). Population-based surveillance conducted in Zhengding (Hebei, China) and Sanjiang County (Guangxi, China) also confirmed GII.3, GII.4, GII.2, GII.6 and GII.17, as the predominant pathogenic genotypes in children < 5-years of age (9).

Vaccination is often considered the most cost-effective way to prevent contagious diseases. In 2016, the World Health Organization stated that the development of an NoV vaccine should be considered an absolute priority (10). Several techniques have been applied to NoV vaccine development, including non-replicating virus-like particles (VLPs), P particles, and recombinant adenoviruses (11). VLPs are virus-genome-free particles that resemble the corresponding authentic viruses in size and shape. They are incapable of infection, but still effective in mounting immune responses (12). They are typically produced by recombinant baculovirus, mammalian cells, *Escherichia coli* (*E. coli*), *Pichia pastoris* (yeast), or plants (13). *Pichia pastoris* is a eukaryotic expression system that is more likely to produce naturally folded VLPs with post-translational modifications than *E. coli*, which indicates a step forward in VLPs production and represents an easily scaled up and cost-effective production system, making it more feasible for commercialized manufacturing (14, 15).

Vaccines against NoVs are likely to face obstacles similar to those faced by seasonal influenza vaccines: new influenza epidemic strains arise every 1 to 2 years due to the general antigenic drift and shift (16). Despite the global surveillance of influenza epidemics around the world, mismatch between circulating strains and prophylactic vaccines occurs occasionally, which may lead to suboptimal vaccine effectiveness (17), and periodical update of vaccine seed strains is costly and time-consuming. Analogously, the diversity of NoV epidemic strains is also one of the major impediments in the development of cross-protective vaccines. Thus, multivalent NoV vaccines that cover both genogroups and antigenically novel GII.4 variants are considered indispensable. Based on the epidemiological status and future trends, we have formulated a *Pichia pastoris* VLP-based 6-valent NoV vaccine to broaden immune protection against human NoVs. Apart from the typical Norwalk GI.1 and essential GII.4 (Hong Kong 2014 strain), we additionally chose GII.P16-GII.2, GII.3 (Hong Kong 2014 strain), GII.6 (Guangdong 2016 strain), GII.17 (Hong Kong 2014 strain) as constituents. Our results have demonstrated that no immunological interference exists among the component antigens. Strain-specific humoral and T-cell responses without mutual immunogenicity inhibition indicate a viable strategy to combine six heterogeneous NoV VLPs together, which sheds light on the development of broad-spectrum NoV vaccines.

## Materials and Methods

### Construction of Recombinant Yeast Expression Vectors

VP1 genes of GI.1 Norwalk strain (GenBank ID: NP_056821.2) (18), Env/CHN/2016/GII.P16-GII.2/BJSMQ (GenBank ID: NC_039476.1), Hu/HKG/2014/GII.3/CUHK-NS-227 (GenBank ID: AHZ12738.1), Hu/GII.4/Hong Kong/CUHK6080/2012/CHN (GenBank ID: KC631827.1) (19), Hu/GII.6/016Q04/ZS/GD/CHN/2016 (GenBank ID: KY407216.1), and Hu/HKG/2014/GII.17/CUHK-NS-491 (GenBank ID: AKB94545.1) (18) were codon-optimized for yeast expression and synthesized by Beijing Tsingke Biotechnology Co., Ltd. They were subcloned onto pPink-hc (Invitrogen, USA) vector with engineered EcoRI and KpnI sites at both ends, respectively. The recombinant plasmids pPink-HC-GI.1, pPink-HC-GII.2, pPink-HC-GII.3, pPink-HC-GII.4, pPink-HC-GII.6, and pPink-HC-GII.17 were identified using EcoRI, KpnI, MfeI, and PvuI enzyme digestion. Approximately 5-10 µg of AflII-linearized plasmids were transformed into *Pichia pastoris* strains (Invitrogen, USA) using an electroporator (Bio-Rad, USA) under a condition of 1.5 kV, 5 ms. Positive colonies were selected from the PAD plates and confirmed *via* PCR and Western blot.

### Preparation of Yeast Extract

Positive clones were selected from PAD plates and cultured in 5 ml BMGY at 220 rpm, 28°C for approximately 30 h, then 1-2 ml solution was transferred to 500 ml BMGY and inoculated under the same conditions until OD600 reached 2-6. Fungi were harvested and added to 100-150 ml BMMY medium, methanol was supplemented every 24 h to reach a final concentration of 1%. After 3-5 days, fungi were collected *via* centrifugation and resuspended in 0.15 M PBS containing 1 mM PMSF, homogenized at 4°C, 1500 bar, and supernatants were then collected *via* centrifugation at 12,000 rpm for 30 min. 7% PEG6000 and 0.2 M NaCl were added to concentrate the supernatants overnight. Concentrated supernatants were centrifuged, the precipitate was resuspended in 0.15 M PBS and dissolved at 4°C overnight. The next day, the pellets were centrifuged at 12,000 rpm for 30 min, and the supernatants were retained.

### Sucrose Density Gradient Centrifugation (SDGC)

Yeast extract supernatant was preliminarily subjected to 20% sucrose cushion ultra-centrifugation at 27,000 rpm for 4 h. Pellets were resuspended in 0.15 M PBS, dissolved at 4°C overnight, layered onto 10-50% sucrose gradients for ultracentrifugation at 39,000 rpm for 3 h (Beckman Coulter, USA). Twelve fractions were taken from top to bottom and analyzed for VLPs content using SDS-PAGE. The original pellet buffer was replaced with PBS through Amicon Ultra-15 (Millipore, USA) centrifugation. Purified VLPs were quantified using the Pierce™ BCA Protein Assay Kit (Thermo Fisher Scientific, USA).

### SDS-PAGE and Western Blot

The VLP samples (5-10 µg) were mixed with 5 × loading buffer, boiled for 10 min, and then were subjected to 12% SDS-PAGE. The gel was stained with Coomassie brilliant blue G250 (Solarbio, China). The purity of VLP protein was analyzed by Image Lab (Bio Rad, USA) software. VLPs with purity greater than 95% were used for mice immunization.

For western blot analysis, the VLP samples were fractionated by SDS-PAGE and then were transferred to PVDF membrane. The membranes were blocked with 5% skim milk for 2 h at room temperature and then were incubated with mouse anti-VP1 monoclonal antibody at 1:5000 dilution overnight at 4°C. Monoclonal antibodies of GII.17 and GII.4 were purchased (GeneTex, USA), while GI.1, GII.2, GII.3, and GII.6 antibodies were customized (GenScript Biotech Corporation, China). Membranes were then incubated with horseradish peroxidase (HRP)-conjugated goat anti-mouse antibody at 1:10000 dilution (Abcam, UK) for 1 h at room temperature. Bands were developed using Chemiluminescent Assay Kit (GenStar, China).

### Negative-Stain Electron Microscopy

The VLPs were subjected to negative staining with 2% phosphotungstic acid, transmission electron microscopy was performed using a Hitachi HT7700 transmission electron microscope (Hitachi, Japan) operated at 80 kV to analyze the shape and integrity of the VLPs.

### Mice Immunization

Female BALB/c mice (6-8 weeks old) were purchased from Vital River Laboratory Animal Technology Co., Ltd (Beijing China). For the dose-escalation study, mice were divided into six groups (five mice/group) as follows: (1) PBS + Alum; (2) 6 µg Hexa-VLPs + Alum; (3) 15 µg Hexa-VLPs + Alum; (4) 30 µg Hexa-VLPs + Alum; (5) 60 µg Hexa-VLPs + Alum; (6) 60 µg Hexa-VLPs. The dose of alum (Invitrogen, USA) was 500 µg per mouse. Mice were immunized intramuscularly (IM) on days 0, 14 and 28.

**Figure 1:**
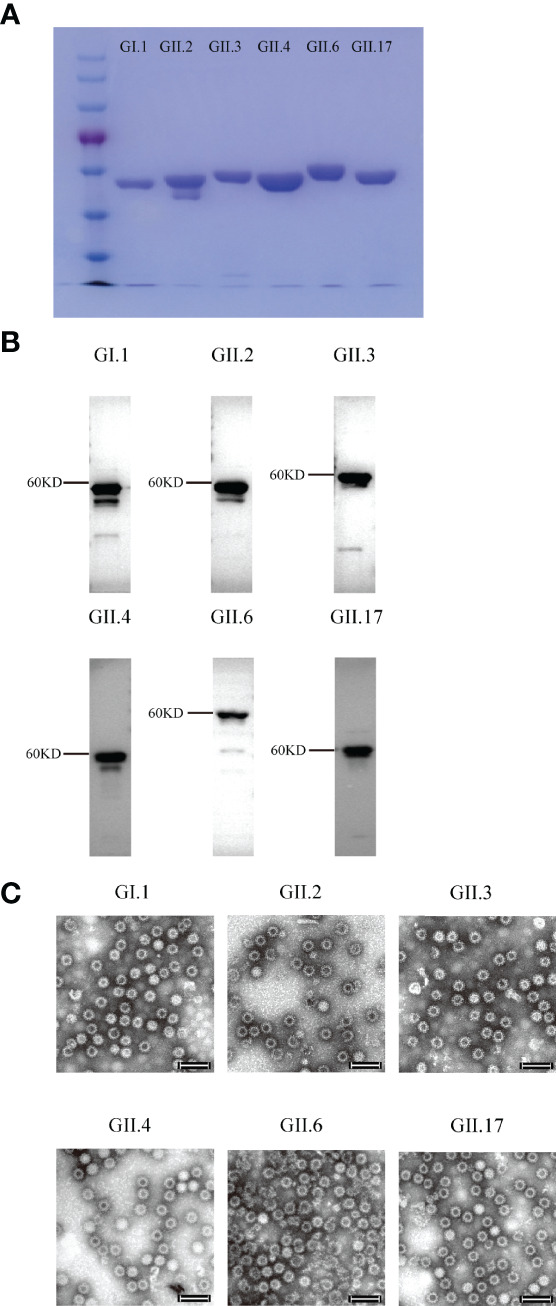
SDS-PAGE, Western blot, and electron microscopy images of the purified norovirus (NoV) virus-like particles (VLPs). **(A)** SDS-PAGE imaging. **(B)** Western blot imaging. **(C)** GI.1, GII.2, GII.3, GII.4, GII.6 and GII.17 VLPs. VLPs were subjected to negative staining with 2% phosphotungstic acid, transmission electron microscopy was performed (Hitachi HT7700 transmission electron microscope, Japan) at 80 kV. Bar = 100 nm.

To compare Hexa-VLPs and monovalent vaccine, mice were divided into ten groups (five mice/group) as follows: (1) PBS + Alum; (2) 5 µg GI.1 + Alum; (3) 5 µg GII.2 + Alum; (4) 5 µg GII.3 + Alum; (5) 5 µg GII.4 + Alum; (6) 5 µg GII.6 + Alum; (7) 5 µg GII.17 + Alum; (8) 1^st^ Hexa-VLP group: 30 µg Hexa-VLPs + Alum; (9) 2^nd^ Hexa-VLPs group: 30 µg Hexa-VLPs + Alum; (10) 3^rd^ Hexa-VLP group: 30 µg Hexa-VLPs + Alum. Mice in 1^st^ Hexa-VLP group were immunized on day 0. Mice in 2^nd^ Hexa-VLPs group were immunized on days 0 and 28. Mice in monovalent groups, PBS group and 3^rd^ Hexa-VLPs groups were immunized on days 0, 14 and 28. The dose of alum (Invitrogen, USA) was 500 µg per mouse. The expression of 2 w, 4 w, 6 w, etc. there-in-after refer to the time point corresponding to the time axis in **Figures 1A**, **4A**.

Blood samples were collected 2 days before each immunization and every 2 weeks after the last inoculation. Blood samples were then placed under 37°C for 15 min, and sera were separated by centrifugation at 4,000 rpm for 10 min and then 12,000 rpm for 15 min. Then sera were stored at -80°C till tested.

### Enzyme-Linked Immunosorbent Assay (ELISA)

VLPs-specific total IgG and IgG1/IgG2a subclass antibodies were detected using ELISA as described elsewhere in detail (20). In brief, sera were 2-fold diluted with an initial dilution of 1:500 fold and incubated on NoV VLPs-coated plates. Plates were then incubated with HRP-conjugated anti-mouse IgG (Abcam, UK) at 1:50,000 dilution, or HRP-conjugated anti-mouse IgG1/IgG2a antibodies (Southern Biotech, USA) at 1:5,000 dilution, respectively, followed by 3,3’,5,5’-tetramethylbenzidine (TMB) substrate (NCM Biotech, China) addition. The optical density was measured at 450 nm using a microplate spectrophotometer (Agilent, USA). The mean endpoint titers of the individual sera were determined as the reciprocal of the highest serum dilution, giving an OD above the set cut-off value. For cross-reactive binding Ab responses, the sera were diluted to 1:1500.

### VLP-Mucin Binding Blockade Assay

The ability of antisera to block VLPs interaction with histo-blood group antigens (HBGAs) was assessed using a well-established blockade assay, in which pig gastric mucin (PGM) type III (Yuanye Bio-Technology Co., Ltd, China) was used as the source of HBGAs (21, 22). Briefly, 100 μl PGM with concentration of 10 μg/ml was coated on an ELISA plate. For homogenous blockade assay, individual sera were 2-fold diluted with an initial dilution of 100-fold, an equal volume mixture of NoV VLPs and serially diluted serum samples was incubated for 1 h and 15 min at room temperature and added to porcine stomach mucin pre-coated plates. The bound VLPs were detected using NoV VLP type-specific antibody, followed by HRP-conjugated goat anti-rabbit IgG (Abcam, UK) and TMB substrate incubation. The OD450 was measured as described above. The maximum binding was determined using VLPs without mouse sera. The blocking titer 50 (BT50) was expressed as the reciprocal of the highest serum dilution blocking 50% of the maximum VLP binding. An arbitrary titer, BT50 of 50, was assigned to samples with <50% blocking index at the lowest serum dilution of 1:100. 12 w sera from 3^rd^ monovalent VLP immunized mice were assayed for cross-blocking antibody response. Sera were diluted 100-fold and incubated with the other 5 heterologous VLPs that were included in Hexa-VLPs, the following procedures were performed as described above.

### ELISPOT Assay

NoV-specific T-cell responses were determined by the ability of VLPs to induce *ex vivo* IFN-γ, TNF-α, IL-2, and IL-4 production using the ELISPOT assay. The BALB/c mice were euthanized after 12 w. The spleens were isolated, mashed, and treated with erythrocyte lysis solution. Multiscreen HTS-IP filter plates (Millipore, USA) were coated with anti-mouse IFN-γ, TNF-α, IL-2, and IL-4 antibodies, according to the manufacturer’s instructions (Mabtech, Sweden). The resuspended splenocytes were then filtered through 70 µm tissue screen and plated at a density of 6×10^5^ cells/well. The VLPs were diluted in RPMI 1640 medium to a final concentration of 10 µg/ml and added to the plates. A negative control (1640 medium) and positive control (cell stimulation cocktail, Thermo Fisher Scientific, USA) were added to each assay. The plates were incubated for 48 h at 37°C and 5% CO2, after which the spots were developed by adding biotinylated anti mouse IFN-γ, TNF-α, IL-2, IL-4 antibodies, HRP-conjugated streptavidin, and TMB substrate. Spots were counted using an Immuno-spot^®^ analyzer (Cellular Technology Ltd., USA). The results are expressed as mean spot-forming cells (SFC)/6 х10^5^ cells for each VLP or control.

### Ethics Statement

All animal studies were approved by the Institutional Animal Care and Use Committee at Tianjin Medical University (ID: TMUaMEC 2021001), and the animals were cared for in accordance with the institutional guidelines.

### Statistical Analysis

The Prism 8.4.2 software (GraphPad, USA) was used to draw graphs and perform statistical analyses. Differences between groups were analyzed using one-way analysis of variance (ANOVA) with Tukey’s adjustment. For every test, statistical significance was indicated as *p < 0.05, **p < 0.01, ***p < 0.001, and ****p < 0.0001.

## Results

### Dose-Dependent Binding Antibody Responses

Six VLPs were produced from the *Pichia pastoris* expression system and identified using Western blot and electron microscopy (**Figures 1A–C**). To determine the optimal inoculation dosage and investigate the immunological effect of Hexa-VLPs, the mice were divided into 6 groups and immunized with different dosages of Hexa-VLPs plus alum through the intramuscular route.

**Figure 2:**
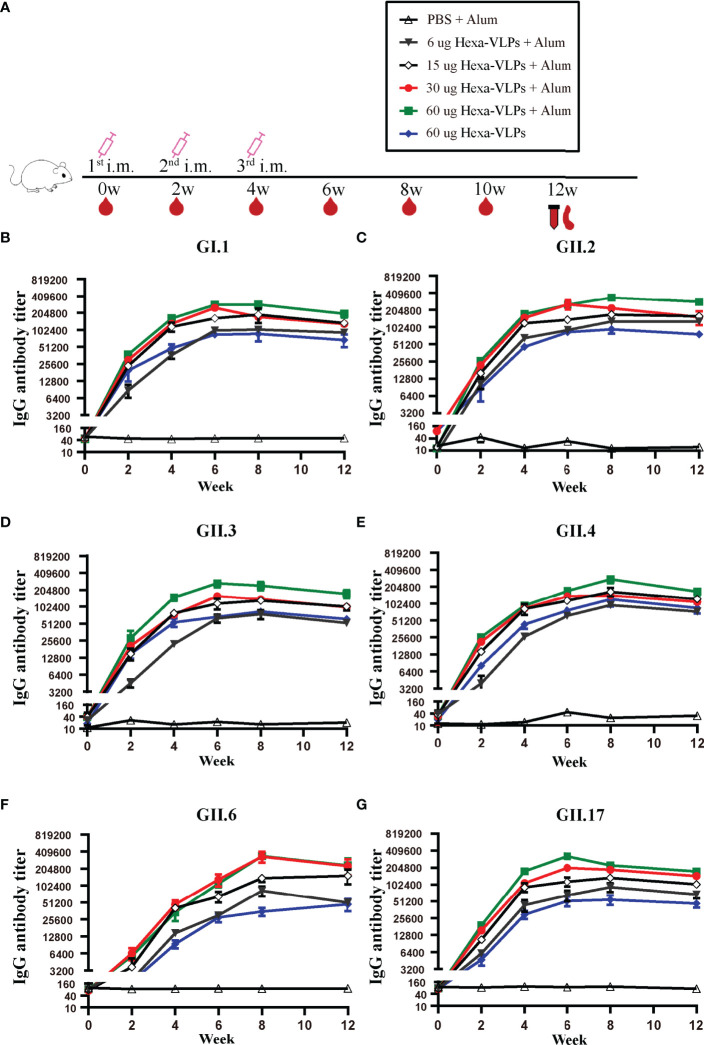
NoV genotype-specific IgG antibody responses induced by diverse Hexa-VLPs dosage. **(A)** Immunization schemes of the study groups. BALB/c mice (n = 5 per group) were immunized intramuscularly (IM) with PBS, 6 µg, 15 µg, 30 µg, 60 µg Hexa-VLPs with 500 µg alum or 60 µg Hexa-VLPs without alum. Each regimen consisted of three shots at day 0, day 14 and day 28. Blood samples were collected 2 days before each immunization and every 2 weeks after the last inoculation. Binding IgG against **(B)** GI.1, **(C)** GII.2, **(D)** GII.3, **(E)** GII.4, **(F)** GII.6 and **(G)** GII.17 VLPs were detected using ELISA. Sera were 2-fold diluted with an initial dilution of 500-fold, endpoint titers of individual serum were determined as the reciprocal of the highest serum dilution giving an OD450 above the cut-off value. All data were shown as means ± SEM (standard error of mean). Statistical differences at 4 w, 8 w, 12 w were analyzed using one-way analysis of variance (ANOVA) with Tukey’s adjustment.

Each regimen consisted of three shots at 14-day intervals (**Figure 2A**). Sera from the PBS + Alum group only showed baseline reactivity against six VLPs, and the Hexa-VLPs immunized mice displayed robust genotype-specific IgG responses (**Figures 2B–G**). For every group, the tendency of binding IgG significantly increased at 2 w and peaked at 6-8 w, then slightly waned at 12 w post vaccination. At 4 w, geometric mean titers (GMT) of GI.1/GII.2/GII.3/GII.4/GII.17-binding IgG induced by 6, 15, 30, and 60 µg Hexa-VLPs increased in a dose-dependent manner. **Table 1** lists the GMT of the dose-escalation study at 4 w, 8 w and 12 w. The GII.6-reactive antibody levels of the 60 µg Hexa-VLPs + Alum group were mildly lower than those of the 15 µg Hexa-VLPs + Alum and/or 30 µg Hexa-VLPs + Alum groups at 2-6 w, yet restored to a dose-dependent tendency at 8 and 12 w. The 60 µg Hexa-VLPs group displayed significantly lower GMTs than the 60 µg Hexa-VLPs + Alum group (p < 0.01).

**Table 1:** 4 w, 8 w and 12 w GMT enumeration of dose-escalation study.

Group	GI.1	GII.2	GII.3	GII.4	GII.6	GII.17
PBS + Alum	45; 50; 50*	13; 13; 15	16; 16; 18	14; 23; 29	85; 85; 89	94; 92; 72
6 µg Hexa-VLPs + Alum	34,830; 99,642; 90,718	63,268; 125,646; 124,371	21,671; 70,049; 51,127	25,807; 92,167; 71,520	141,15; 77,401; 50,926	37,732; 85,719; 65,001
15 µg Hexa-VLPs + Alum	108,325; 168,131; 130,670	117,092; 167,515; 157,385	75,639; 127,197; 98,251	75,287; 157,777; 122,733	39,759; 133,151; 129,283	86,687; 134,073; 100,411
30 µg Hexa-VLPs + Alum	130,596; 174,125; 125,955	146,285; 213,570; 25,485	71,967; 137,581; 99,415	82,631; 141,935; 107,893	44,545; 295,109; 183,756	108,061; 189,121;145,545
60 µg Hexa-VLPs + Alum	158,733; 270,206; 189,634	171,170; 329,447; 283,477	143,966; 228,959; 164,646	92,891; 264,211; 159,860	28,562; 335,154; 189,129	172,146, 222,189;171,513
60 µg Hexa-VLPs	45,603; 77,080; 60,887	45,426; 88,798; 74,415	50,193; 80,533; 60,504	41,345; 108,483; 79,216	8,782; 33,173; 40,700	27,805; 50,021; 44,052

*The three figures from left to right correspond to GMT at 4 w,8 w and 12 w, respectively.

### Broad Blockade Antibody Responses

**Figure 3:**
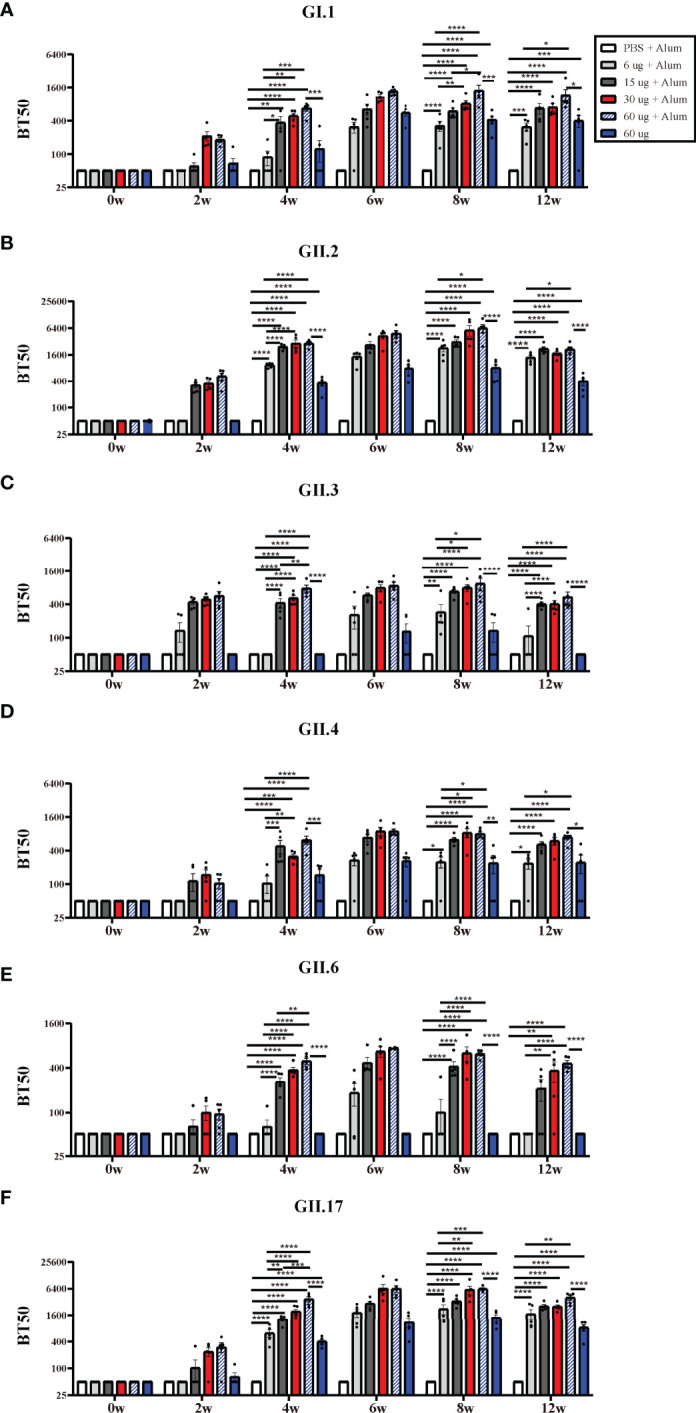
NoV genotype-specific blockade antibody responses induced by diverse Hexa-VLPs dosage. Individual mouse sera were analyzed for blockade antibody titers using the VLP-mucin binding blockade assay. Blockade antibody against **(A)** GI.1, **(B)** GII.2, **(C)** GII.3, **(D)** GII.4, **(E)** GII.6 and **(F)** GII.17 VLPs were detected *via* ELISA. Blocking titer 50 (BT50) was expressed as the reciprocal of the highest serum dilution blocking 50% of the maximum VLP binding. An arbitrary titer, BT50 of 50, was assigned to samples with <50% blocking index at the lowest serum dilution of 1:100. All data were shown as means ± SEM. Statistical differences at 4 w, 8 w, 12 w were analyzed using one-way ANOVA test with Tukey correction for multiple testing. p < 0.05 (*) was considered statistically significant; p < 0.01(**); p < 0.001(***); p < 0.0001(****).

The recognition that HBGA expression is important as a viral attachment factor led to the concept that antibodies which block VLP binding to HBGAs could have neutralizing activity and that HBGA-blocking antibody levels could serve as a surrogate for neutralizing antibody levels (23, 24). High levels of serum blocking antibodies correlate with protection against clinical NoV gastroenteritis (25, 26). Hexa-VLPs at all dosages induced significant type-specific blocking antibody responses and demonstrated a synchronized trend with the binding antibodies. In general, the BT50s against GI.1, GII.3, GII.4, GII.6, and GII.17 in the 30 µg Hexa-VLPs + Alum group revealed comparable values with the 60 µg Hexa-VLPs + Alum group, and higher than those in the 6 µg Hexa-VLPs + Alum group at 4 w and 8 w (p < 0.05), respectively (**Figures 3A, C–F**). As for GII.2, antisera of 4 w mice still maintained this trend, while no significant differences were detected between the 6 µg Hexa-VLPs + Alum group (GMBT50: 2,072) and the 30 µg Hexa-VLPs + Alum group (GMBT50: 4,834) at 8 w (**Figure 3B**). At 4 w, the 15 µg Hexa-VLPs + Alum group also showed superior blockade antibody titers against six VLPs, albeit only higher than that of the 6 µg + Alum group (p < 0.05). Although there were no statistical differences between the 15 µg Hexa-VLPs + Alum group and the 30 µg Hexa-VLPs + Alum group, dose-dependent GMT was still elicited between them at 6 w and 8 w. The 60 µg Hexa-VLPs group still displayed lower levels of BT50s against 6 VLPs than the 60 µg Hexa-VLPs + Alum group (p < 0.05), which further highlights the essentiality of adjuvant addition.

Hexa-VLPs is pioneer in inducing robust protective immunity against recombinant GII.P16-GII.2 and GII.17 (Kawasaki 2014) genotypes. 30 µg Hexa-VLPs + Alum elicited a peak GII.P16-GII.2-specific GMBT50 of 4,834 at 4 weeks after the third inoculation, and also triggered a potent GII.17-blocking GMBT50 of 5,818 at 2 weeks after the third vaccination. These data confirmed the dominance of Hexa-VLPs in blocking these two emerging genotypes, which hold the potential to cause major epidemics in the future.

### VLPs-Specific T-Cell Responses

To measure the cellular immune responses elicited by Hexa-VLPs, T-cell responses against six VLPs were monitored in immunized mice *via* ELISPOT assays at the end of the experiment (**Figure S1**). The mean number of cytokine-producing splenocytes in the 30 µg Hexa-VLPs + Alum and 60 µg Hexa-VLPs + Alum groups increased after immunization, especially for IL-2 and IL-4 (**Figures S1C, D**). As for the IFN-γ and TNF-α, GI.1-, GII.2-, GII.6- and GII.17-specific spot tended to be induced more distinctly (**Figures S1A, B**). Protective immunity against NoV might be partially dependent on the activation of T-cell immunity (27), and our data support a durable cellular immune response induced by Hexa-VLPs.

### Comparison of Hexa-VLPs and Monovalent Vaccines

**Figure 4:**
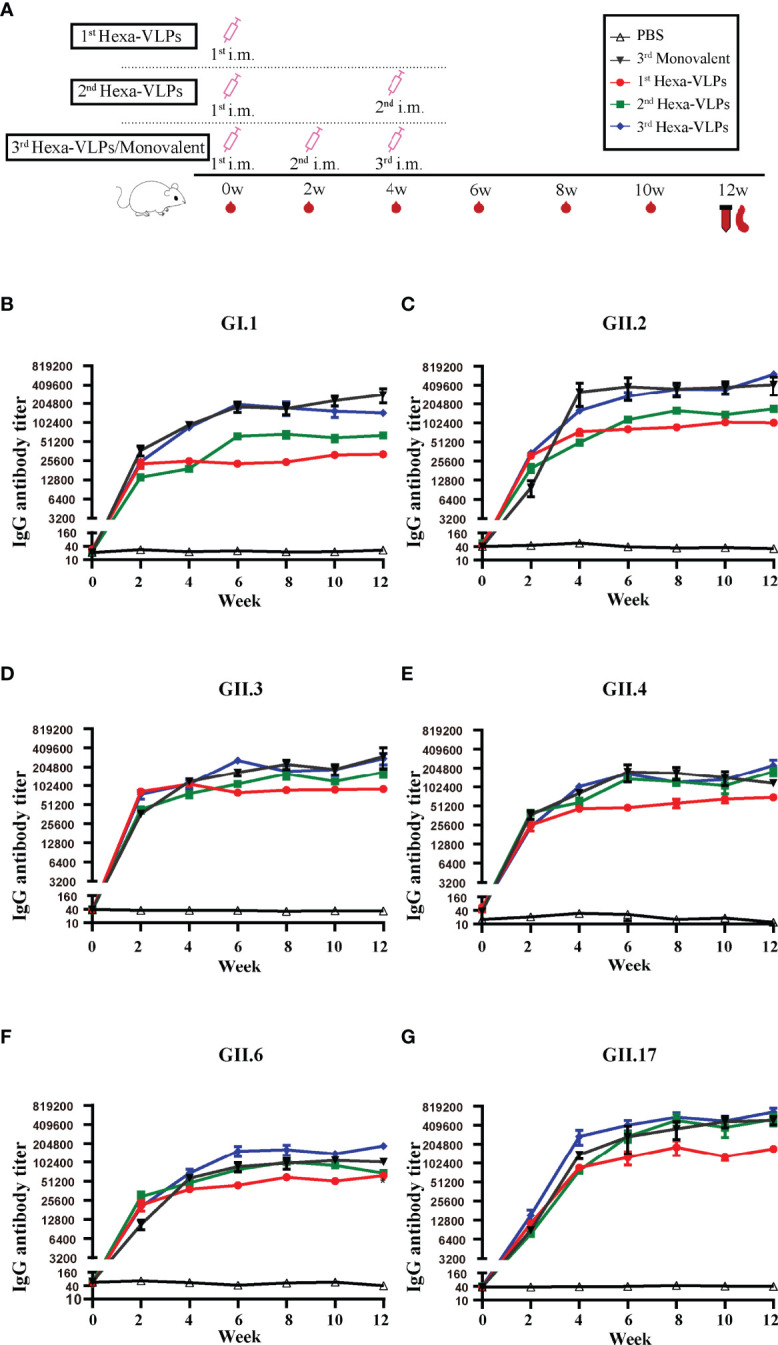
Total IgG responses of different immune regimens. **(A)** Immunization schemes of mice. BALB/c mice (n = 5 per group) were immunized IM. The control group, six monovalent groups and 3^rd^ Hexa-VLPs group were immunized at day 0, day 14 and day 28 with PBS, 5 µg GI.1/GII.2/GII.3/GII.4/GII.6/GII.17 or 30 µg Hexa-VLPs (each VLPs accounts for 5 µg), respectively. For the 1^st^ Hexa-VLPs group, 30 µg Hexa-VLPs were inoculated at day 0, while the 2^nd^ Hexa-VLPs group at day 0 and day 28. All VLPs were adjuvanted with equal volume of 500 µg alum. Blood samples were collected 2 days before each immunization and every 2 weeks after the last inoculation. Mice were euthanized at 12 w for spleen isolation. Binding IgG against **(B)** GI.1, **(C)** GII.2, **(D)** GII.3, **(E)** GII.4, **(F)** GII.6 and **(G)** GII.17 VLPs were detected using ELISA. Sera dilution and endpoint titer calculation were consistent with **Figure 2**. All data were shown as means ± SEM. Statistical differences at 4 w, 8 w, 12 w were analyzed using one-way ANOVA test with Tukey correction for multiple testing.

Based on these results, a medium-dose vaccine (30 µg Hexa-VLPs plus 500 µg alum) was able to induce ideal immunity and thus chosen for further studies. We compared Hexa-VLPs with six monovalent VLPs (**Figure 4**). NoVs-specific IgG were maintained at the baseline level in all control animal sera (PBS), yet significantly increased at 2 w and peaked at 4-6 w post VLPs vaccination (**Figures 4B–G**). No significant differences (p > 0.05) were observed when comparing VLPs-specific IgG responses of 3^rd^ Hexa-VLPs with 3^rd^ monovalent VLP groups at 4, 6 and 12 w, except for GII.6-binding IgG at 12 w, which showed higher endpoint titers in 3^rd^ Hexa-VLPs immunized mice (p < 0.05) (**Figures 4B–G**). Generally, immunological interference should be considered for multi-valent NoV vaccines (20), whereas Hexa-VLPs induced an equal magnitude of IgG antibodies against six NoVs compared with monovalent vaccines. At 6 w and 8 w, 6 VLPs-specific GMT presented an explicit boost-dependent tendency; in general, the 3^rd^ Hexa-VLPs strategy triggered the most robust binding antibody responses, and the 2^nd^ Hexa-VLPs strategy elicited a suboptimal effect. Thus, a booster injection would consolidate the immune effect of the priming inoculation, supporting the feasibility of booster immunization.

Owing to the homology of the six chosen VP1 sequences, we found ubiquitous cross-reactivity among the six VLP strains (**Figure S2**). General statistical differences existed between the PBS group and all of the monovalent vaccine immunized groups against six VLPs (p < 0.05, asterisks are not marked on the figure). Cross-reactive antibodies against GII.17 VLP were comparable among the GII.2, GII.3, GII.4, and GII.17 groups (p > 0.05) (**Figure S2F**), but for the other five VLPs, the most robust binding was still induced by the corresponding monovalent group and the 3^rd^ Hexa-VLPs group (**Figures S2A–E**). This is consistent with the conclusion that 3^rd^ Hexa-VLPs induced comparable levels of type-specific antibody responses to six monovalent vaccines (p > 0.05).

**Figure 5:**
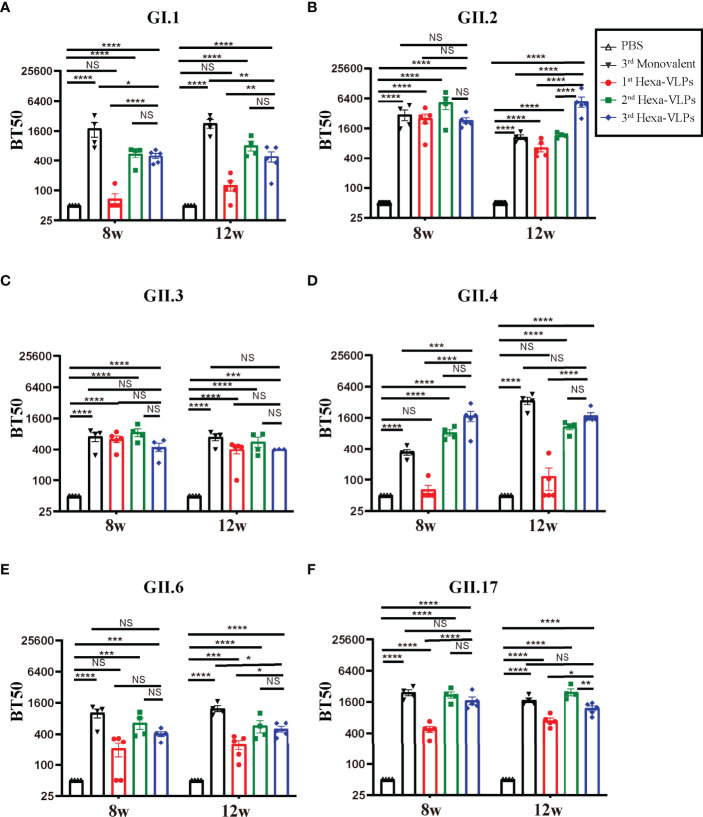
Blockade assay of antisera from different immune regimens. Individual mouse sera collected at 8 w and 12 w were analyzed. Blockade antibody against **(A)** GI.1, **(B)** GII.2, **(C)** GII.3, **(D)** GII.4, **(E)** GII.6 and **(F)** GII.17 VLPs were detected using the VLP-mucin binding blockade assay. BT50 was expressed as the reciprocal of the highest serum dilution blocking 50% of the maximum VLP binding. An arbitrary titer, BT50 of 50, was assigned to samples with <50% blocking index at the lowest serum dilution of 1:100. All data were shown as means ± SEM. Statistical differences were analyzed using one-way ANOVA test with Tukey correction for multiple testing. p < 0.05 (*) was considered statistically significant; p < 0.01(**); p < 0.001(***); p < 0.0001(****); NS, no significance.

According to the tendency of the humoral immune responses depicted above (**Figures 2**-**4**), the humoral responses of 8 w antisera were representative of plateau values, whereas a mild downward trend began to emerge at 12 w. Hence, to determine the magnitude and durability of the VLPs-specific blockade antibody responses, sera at 8 w and 12 w were assayed against a panel of VLPs (**Figure 5**). The 2^nd^ and 3^rd^ Hexa-VLPs groups triggered robust blocking responses against six VLPs when compared with the PBS group (p < 0.001). At 12 w post-vaccination, antisera from the 3^rd^ Hexa-VLPs group showed higher BT50 against GII.2 than the GII.2 monovalent group (**Figure 5B**), as did 8 w sera against GII.4 (**Figure 5D**) (p < 0.001). Only the GI.1-blocking antibody level triggered by 3^rd^ Hexa-VLPs decreased (**Figure 5A**) compared with the 3^rd^ GI.1 group (p < 0.05), whereas all the other antisera from the 3^rd^ Hexa-VLPs group elicited comparable genotype-specific blocking responses with the corresponding 3^rd^ monovalent vaccine (p > 0.05). Overall, peak blockade antibodies against GII.2, GII.4 and GII.17, which were elicited by either 2^nd^ or 3^rd^ Hexa-VLPs, were more advantageous than those against GI.1, GII.3 and GII.6. **Table 2** lists the GMBT50 of different regimens at 8 w and 12 w. We deem that the magnitude of genotype-specific blockade antibody responses triggered by the 2^nd^ and 3^rd^ Hexa-VLPs remained steady throughout the entire test period. Hence, sequential immunization with two or three shots of Hexa-VLPs presented a more advantageous immune effect than the single-dose regimen. There were no detectable cross-blocking antibodies induced by 3^rd^ monovalent VLP immunized antisera, which further confirms the necessity of our novel multivalent vaccine (**Figure S3**).

**Table 2:** 8 w and 12 w GMBT50 enumeration of different regimens.

Group	GI.1	GII.2	GII.3	GII.4	GII.6	GII.17
PBS	50; 50^#^	50; 50	50; 50	50; 50	50; 50	50; 50
3^rd^ Monovalent*	1,498; 2,126	2,734; 1,054	656; 667	334; 3,273	946; 1,217	2,369; 1,716
1^st^ Hexa-VLPs	61; 113	2,213; 623	603; 345	60; 84	196; 202	463;687
2^nd^ Hexa-VLPs	520; 764	4,452; 1,164	823; 522	808; 1,038	599; 523	2,127; 2,385
3^rd^ Hexa-VLPs	487; 415	2,241; 4,946	414; 400	1,538; 1,679	395; 485	1,625; 1,161

^#^The two figures from left to right correspond to GMBT50 at 8 w and 12 w, respectively.

*The data listed in this line are the GMBT50 of monovalent vaccine against homogenous VLPs, cross-blocking data are not shown here.

### Hexa-VLPs Induced Mixed Th1/Th2 Responses

**Figure 6:**
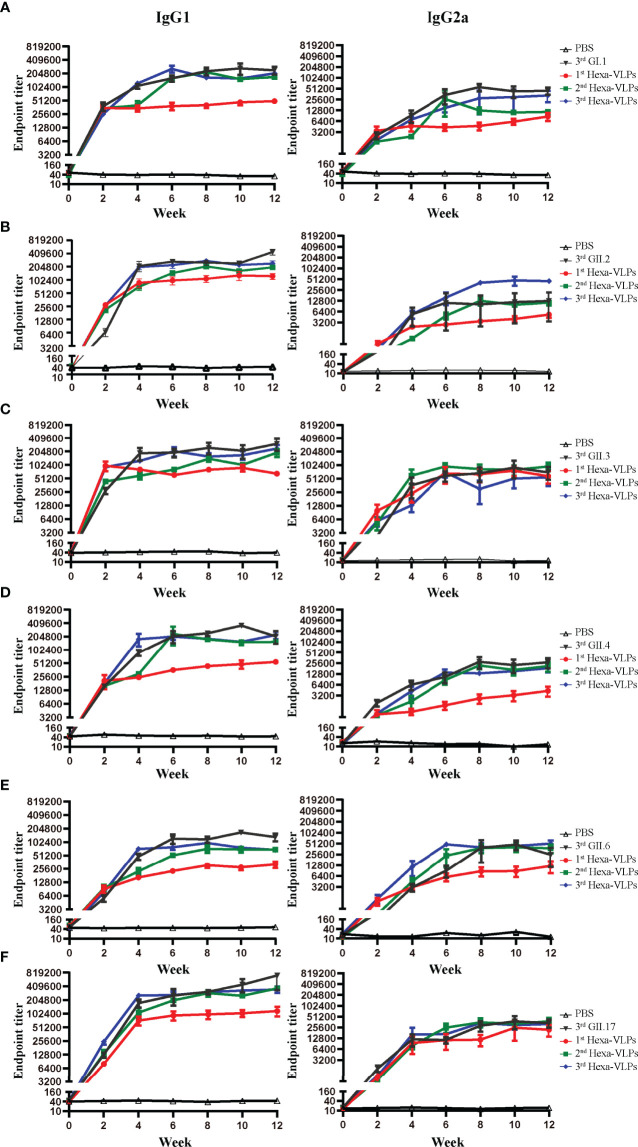
IgG subclass antibody detection of different immune regimens. Isotype profiles were determined with direct ELISA using plates coated with the purified **(A)** GI.1, **(B)** GII.2, **(C)** GII.3, **(D)** GII.4, **(E)** GII.6 and **(F)** GII.17 VLPs. Each column from left to right represents endpoint titers of IgG1, IgG2a, respectively. Sera were 2-fold diluted with an initial dilution of 500-fold, endpoint titers of individual serum were determined as the reciprocal of the highest serum dilution giving an OD450 above the cut-off value. All data were shown as means ± SEM. Statistical differences at 4 w, 8 w, 12 w were analyzed using one-way ANOVA test with Tukey correction for multiple testing.

We further delineated the magnitude of the IgG subclass profile following NoV VLPs vaccination. Each formulation generated high levels of IgG1 and IgG2a antibodies, indicating a mixed Th1/Th2 response (**Figures 6A–F**). In all cases, the tendency of two subclass antibodies against six VLPs significantly increased at 2 w and peaked at 4-6 w post vaccination. 3^rd^ Hexa-VLPs group still displayed higher or equivalent levels of subclass antibody titers to monovalent VLPs (p > 0.05).

It has been documented that IgG1 is mainly promoted by Th2 cells producing IL-4, IL-5, IL-6, IL-10 and IL-13 whereas IgG2a, IgG2b and IgG3 production is mainly promoted by Th1-related cytokines such as IFN-γ, TNF-α, and IL-2 (28, 29). We have performed a more detailed analysis of the isotype profiles with 8 w sera (**Figure S4**). Hexa-VLPs plus alum regimen induced a Th2-skewed humoral immune response as the GMT of IgG1 was significantly higher than that of IgG2a. Only GII.6-specific IgG2a and IgG2b made no statistical difference with IgG1, with GMT of 30,149 and 94,622 respectively. Alum is the most commonly used adjuvant in prophylactic vaccines, and the propensity of alum based adjuvants to induce a Th2-skewed immune response has been widely approved (30, 31)

Accordingly, IFN-γ, TNF-α, IL-2, and IL-4 ELISPOT assays were performed on splenocytes (**Figure S5**). Overall, four cytokines were induced by Hexa-VLPs to a certain extent, especially for IL-2 and IL-4 (**Figures S5C, D**). GI.1-, GII.2-, GII.3- and GII.6-specific IL-4-producing splenocytes were significantly activated by 3-shot Hexa-VLPs vaccination, compared with PBS group (p < 0.01).

## Discussion

As self-assembled, highly immunogenic structures, VLPs have gained popularity in recombinant vaccine development because of their intrinsic characteristics (32–34). A handful of prophylactic VLP-based vaccines are licensed and commercially available: GlaxoSmithKline’s Engerix^®^ (hepatitis B virus) (35), Cervarix^®^ (human papillomavirus) (36), Merck and Co., Inc.’s Recombivax HB^®^ (hepatitis B virus) (37) and Gardasil^®^ (human papillomavirus) (38). Two VLP-based NoV candidate vaccines are also undergoing clinical evaluation in China: (i) a bivalent vaccine against GI.1 and GII.4, which was developed by Lanzhou Institute of Biological Products Co., Ltd., has completed its phase I clinical trial (CTR20192432) with a phase II clinical trial (CTR20211467) in progress; and (ii) a tetravalent recombinant norovirus vaccine against GI.1, GII.3, GII.4, and GII.17, which was developed by Anhui Zhifei Longcom Biopharmaceutical Co., Ltd., is recruiting volunteers for its phase I clinical trials (CTR20191769). Nevertheless, all NoV vaccine candidates so far cannot cover major circulating strains, which means that broader-spectrum NoV vaccines are in urgent demand. Hence, we have launched a 6-valent NoV vaccine using the VLP platform.

In our preclinical study, both the 2-shot and 3-shot regimens induced potent binding antibody responses in mice, which were comparable to those of a VLP-based bivalent vaccine (covering NoV GII.4 and Enterovirus 71) (19) and a tetravalent NoV vaccine (covering GI.1, GI.3, GII.4, GII.12 genotypes) (20). The adenoviral (Ad) vector has also been applied in norovirus vaccine development, and Guo et al. assessed the immune responses of an AdHu5-based monovalent GII.4 vaccine in mice, which elicited a relatively weak GMT post-priming immunization, and mounted to 632,978 at 14 days post the third immunization (39). They further performed a sequential immunization strategy of Ad prime-VLP boost formulation to alleviate the impact of pre-existing anti-AdHu5 immunity. In comparison with the VLP prime-rAd boost and VLP-alone regimens, the rAd prime-VLP boost regimen induced higher GII.4-specific GMT levels (>100,000). It is likely that rAd and NoV VLPs stimulate innate immunity in different ways, leading to the reinforcement of subsequent adaptive immunity (40).

Higher levels of serum antibodies that block viral binding to HBGAs are associated with a lower risk of illness and infection (26). The first-in-human efficacy trial of Takeda’s bivalent norovirus vaccine candidate (TAK-214) reported a characteristic increase in GI.1-and GII.4c-specific HBGA-blocking antibodies at 8 days after a single-dose inoculation, with peak GII.4c-GMT >1,000 and this slightly waned to <1,000 on day 45 (41). GII.4c, i.e., GII.4 consensus VLP, was designed by aligning three genetically-distinct GII.4 capsid protein sequences and selecting the “consensus” amino acid residues at each position (42). A phase I clinical trial (NCT01168401) confirmed that a 50 µg GI.1/50 µg GII.4c regimen could induce a GMEC50 (carbohydrate-binding blockade assay) of 613 (GI.1) and 953 (GII.4c) at day 7 post-priming vaccination (43). A Phase II clinical trial launched by Ghent University (NCT02038907) found that 15 µg GI.1/50 µg GII.4c plus 500 µg alum elicited the best balance of immunogenicity in healthy adults, with peak GMEC50s of 413 (GI.1) and 843 (GII.4c) post vaccination (44). A clinical trial (NCT1609257) of another bivalent NoV VLP vaccine showed that anti-GII.4 HBGA-blocking antibody levels >1:500 was associated with a lower frequency of moderate-to-severe vomiting or diarrheal illness in humans (45). In this study, our Hexa-VLPs elicited comparable HBGA-blocking antibody responses, with a 3-shot regimen producing a GII.4-specific GMT of 821 at 14 days after the last vaccination and remained at an elevated level until 12 w (GMT=554). The peak GMT of GI.1 attained 1,079 simultaneously with GII.4 (**Figure 3**).

The epidemic of GII.17 Kawasaki (46) and recombinant GII.P16-GII.2 strains (47) has attracted extensive attention worldwide, raising concerns about their replacement of the GII.4 strains. The spatiotemporal diffusion analysis of GII.P17-GII.17 strains in Zhoushan Islands during 2013–2018 had already identified Hong Kong GII.17 (Kawasaki) as the epicenter for GII.17 dissemination in China (48), which calls for a focus on non-GII.4 genotypes. In addition, immunity after natural NoV infection is short-lived (<2 years) (49); therefore, the intensity and duration of blockade antibody responses post-vaccination are pivotal. According to our long-term immune response monitoring of 30 µg Hexa-VLPs, the blockade antibody responses against GII.2, GII.3, GII.6, and GII.17 soared synchronously after booster vaccination (4 w), with peak GMBT50s of 610 to 5,818 at 6 or 8 w (**Figure 3**). Our Hexa-VLPs elicited potent and durable blockade antibody responses against six major circulating NoV strains, yet no detectable cross-blocking antibodies were induced by monovalent VLP immunized antisera. There is evidence that VLP-based multivalent NoV vaccination in mice could induce robust hetero-genotype immunity against additional NoVs that were not included in the vaccine mix (50). Phase I clinical trial showed that Takeda’s bivalent NoV vaccine (GI.1/GII.4c) induced a broadly blocking Ab response to both vaccine components and non-vaccine NoV strains, and to two novel GII.4 strains not in circulation at the time of vaccination (43). Comparing with monovalent vaccine, cocktail immunization renders an accumulation of immune effects, thus increases the probability of mounting cross-block antibodies, which were typically more robust within a genogroup than between genogroups (50). Hence, the blocking responses against genotypes and variants not included in Hexa-VLPs or newly emerging NoVs need to be further explored.

Malm et al. confirmed that sequential immunization with genetically distant genotype (i.e., GI.3 + GII.4 prime-GII.17 boost) was successful in inducing high GII.17-specific immune response, while sequential immunization with closely related VLP (i.e., GI.3 + GII.4 1999 prime-GII.17 boost-GII.4 SYD boost) failed to induce strong GII.4 SYD-blocking responses compared to simultaneous immunization. These results indicate that there might be an original antigenic sin (OAS) for closely related antigens, and simultaneous immunization (a cocktail) could avert the problem of immunological interference among component antigens. Malm’s multivalent VLP vaccine showed no immunological inhibition when delivered simultaneously, as binding and blocking antibodies were at an equal level to the responses induced by monovalent VLPs (51). Hexa-VLPs also showed no inhibition of NoV-specific antibody functionality or magnitude when compared with monovalent vaccines (**Figures 4**–**6**).

Previous studies have demonstrated that VLPs effectively promoted the phenotypic maturation of macrophages, which present VLP antigens to naïve CD4^+^ T cells and induce the differentiation of Th0 cells toward Th1 and Th2 phenotypes (52). Accordingly, Hexa-VLPs elicited mixed Th1/Th2 responses signified by high-level IFN-γ, TNF-α, IL-2 and IL-4 positive T-cell activation. Current licensed adjuvants, including alum, are known to preferentially prime Th2-type immune responses (30), and typically induce strong antibody responses characterized by the differentiation of IL-4-producing CD4^+^ T cells and IgG subclass switching to IgG1 (53). Consistently, Hexa-VLPs plus alum elicited Th1/Th2 mixed, yet IgG1 isotype-biased antibody response and optimal IL-4 production (**Figures S1, S5**). Data from the mouse NoV model suggest that T cells are critical for achieving viral clearance (54). Hexa-VLPs are superior for inducing extensive and long-lasting cellular immunity.

Our study has some limitations. Firstly, the heterologous blocking responses against genotypes and variants not included in Hexa-VLPs were not tested. Secondly, we only performed the immunological evaluation of Hexa-VLPs in BALB/c mice, application of other animal models such as non-human primates (NHPs) would provide insight into the usefulness of Hexa-VLPs. In conclusion, by combining an ancestor Norwalk GI.1 strain with five other candidate strains, we have developed a novel 6-valent NoV vaccine to guard against multiple NoV genotypes. Hexa-VLPs broadened the breadth of antibody repertoire on the basis of improving their magnitude and elicited long-term memory T-cell responses. Together, Hexa-VLPs hold the potential to become a broad-spectrum NoV vaccine candidate.

## Data Availability Statement

The datasets presented in this study can be found in online repositories. The names of the repository/repositories and accession number(s) can be found in the article/**Supplementary Material**.

## Ethics Statement

The animal study was reviewed and approved by Institutional Animal Care and Use Committee at Tianjin Medical University(ID: TMUaMEC 2021001).

## Author Contributions

DZ, HL, and WH conceived, designed, and supervised the entire study. LL, JR constructed the vaccines and carried out all immunogenicity evaluations in mice. LL, YW, MX, and JR performed data analysis. YW and DZ wrote the manuscript draft. YG and DZ were responsible for manuscript reviewing and editing. WH, HL, DX, XW, and XZ provided experimental resources and assisted all immunology experiments. All authors contributed to the article and approved the submitted version.

## Funding

This work was chiefly supported by funds from the National Natural Science Foundation of China (32070926, 31870922), and partially supported by the key project of the Natural Science Foundation of Tianjin (20JCZDJC00090).

## Conflict of Interest

Authors DX, XW, XZ, and HL are employed by Chengdu Kanghua Biological Products Co., Ltd.

The remaining authors declare that the research was conducted in the absence of any commercial or financial relationships that could be construed as a potential conflict of interest.

## Publisher’s Note

All claims expressed in this article are solely those of the authors and do not necessarily represent those of their affiliated organizations, or those of the publisher, the editors and the reviewers. Any product that may be evaluated in this article, or claim that may be made by its manufacturer, is not guaranteed or endorsed by the publisher.
