# Supplementary material for: 6-Valent Virus-Like Particle-Based Vaccine Induced Potent and Sustained Immunity Against Noroviruses in Mice
Source: Front Immunol. 2022 May 23;13:906275. doi: 10.3389/fimmu.2022.906275 (PMC9197435; doi:10.3389/fimmu.2022.906275)
Supplement: Supplementary file 1 [file DataSheet_1.docx]

Supplementary Material


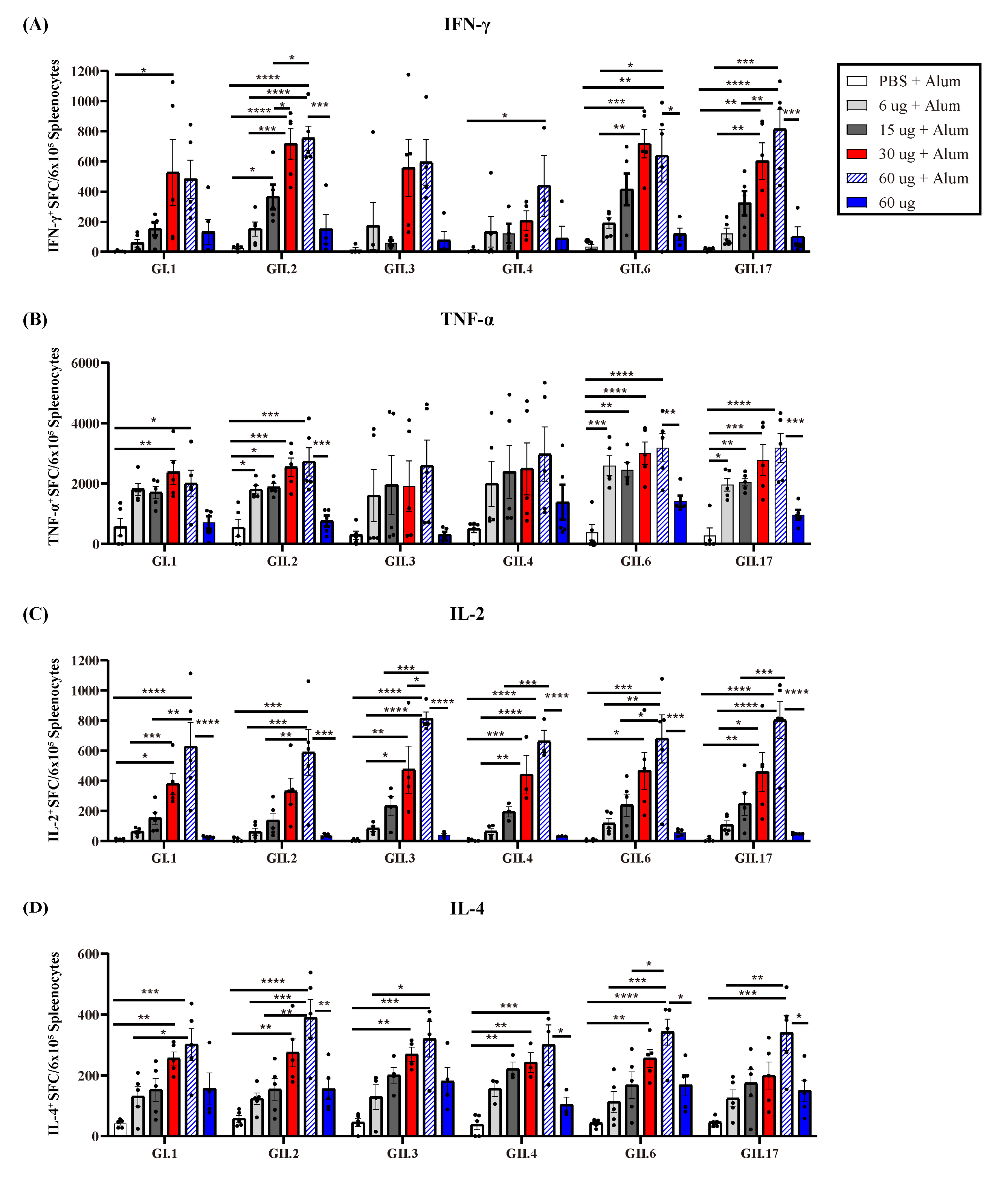


**Supplementary Figure 1.** IFN-γ, TNF-α, IL-2 and IL-4 ELISPOT assays of mouse splenocytes from dose escalation study. BALB/c mice were euthanized at 12 w, splenocytes were isolated and stimulated with 10 ug/ml VLPs. (A) IFN-γ, (B) TNF-α, (C) IL-2 and (D) IL-4 producing cells were quantitated using an ELISPOT reader. Results were expressed as the average number of SFC per 6 х 10^5^ input splenocytes. All data were shown as means ± SEM (n=3-5). Statistical differences were analyzed using One way ANOVA test with Tukey correction for multiple testing. p < 0.05 (*) was considered statistically significant; p < 0.01 (**); p < 0.001 (***); p < 0.0001 (****).


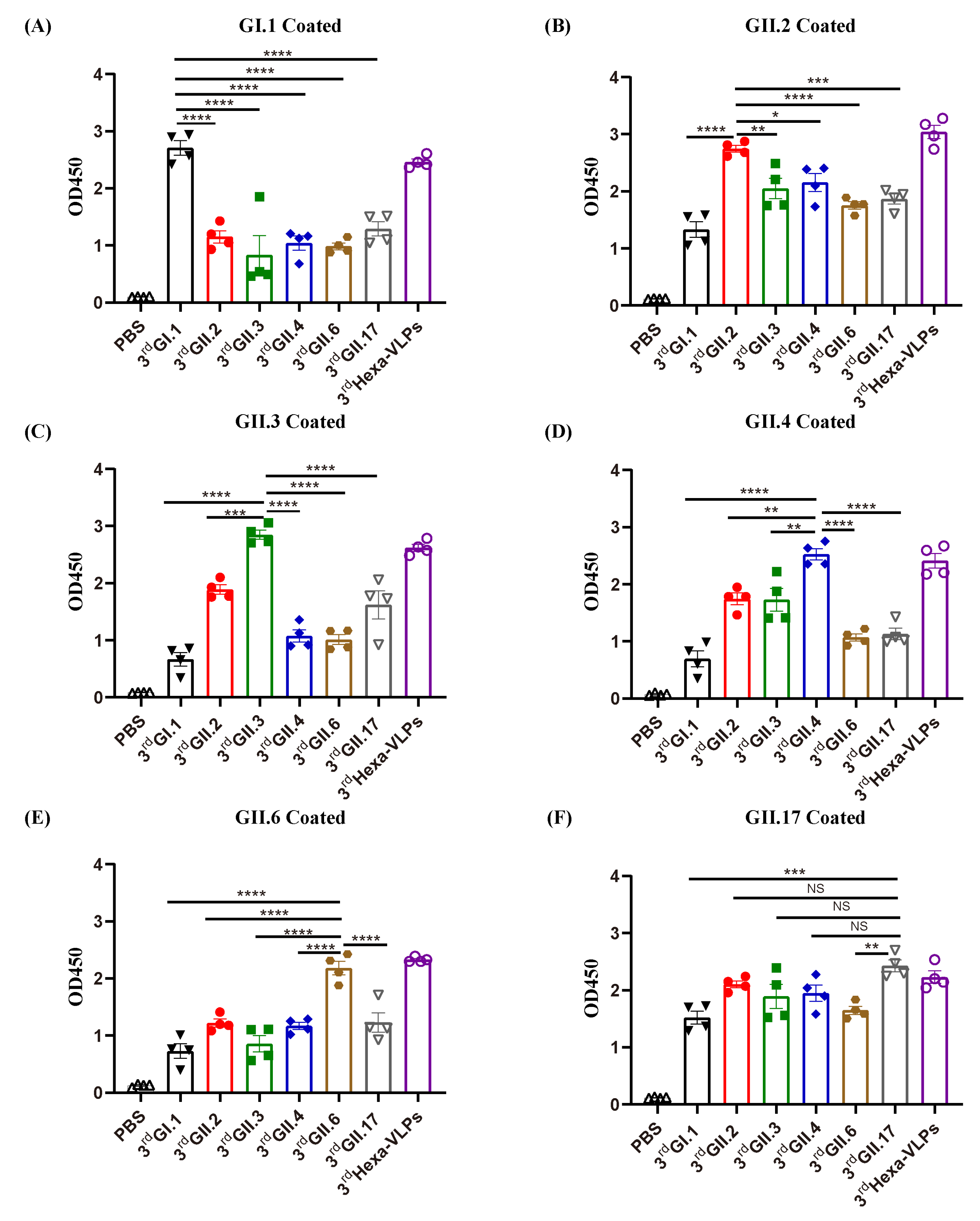


**Supplementary Figure 2.** Cross-reactive antibody detection against a panel of VLPs. 8 w antisera from 30 ug Hexa-VLPs or 5 ug monovalent VLPs immunized mice were diluted by 1,500-fold and performed direct ELISA using plates coated with the purified (A) GI.1, (B) GII.2, (C) GII.3, (D) GII.4, (E) GII.6 and (F) GII.17 VLPs. All data were shown as means ± SEM. Statistical differences were analyzed using One way ANOVA test with Tukey correction for multiple testing. Due to the general statistical differences between PBS group and all of the VLPs immunized groups, asterisk was not marked on the figure. Statistical analyses were performed among monovalent groups that was in consistent with coating VLPs and other five monovalent groups. p < 0.05 (*) was considered statistically significant; p < 0.01 (**); p < 0.001 (***); p < 0.0001 (****).


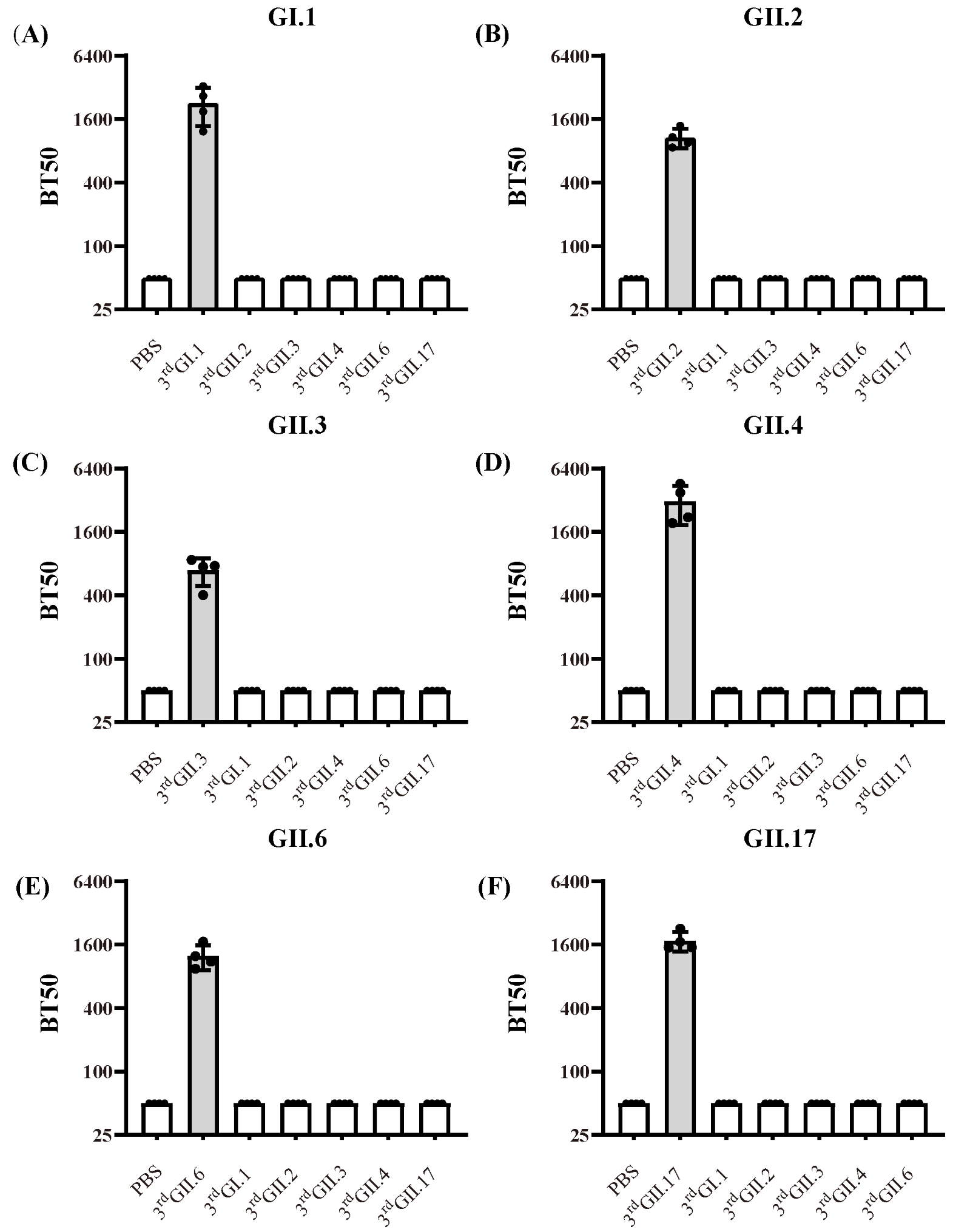


**Supplementary Figure 3.** Cross-blocking antibody responses of 3^rd^ monovalent antisera against heterogenous VLPs. 12 w antisera from 3^rd^ monovalent VLP immunized mice were diluted 100-fold and incubated with the other 5 heterologous VLPs that included in Hexa-VLPs. Cross-blocking antibody responses against (A) GI.1, (B) GII.2, (C) GII.3, (D) GII.4, (E) GII.6 and (F) GII.17 VLPs were detected via HBGA-blocking assays. Blocking titer 50 (BT50) was expressed as the reciprocal of the highest serum dilution blocking 50% of the maximum VLP binding. An arbitrary titer, BT50 of 50, was assigned to samples with <50% blocking index at the lowest serum dilution of 1:100. All data were shown as means ± SEM. BT50 of 3^rd^ monovalent antisera against homogenous VLP were displayed in the corresponding graphs for reference.


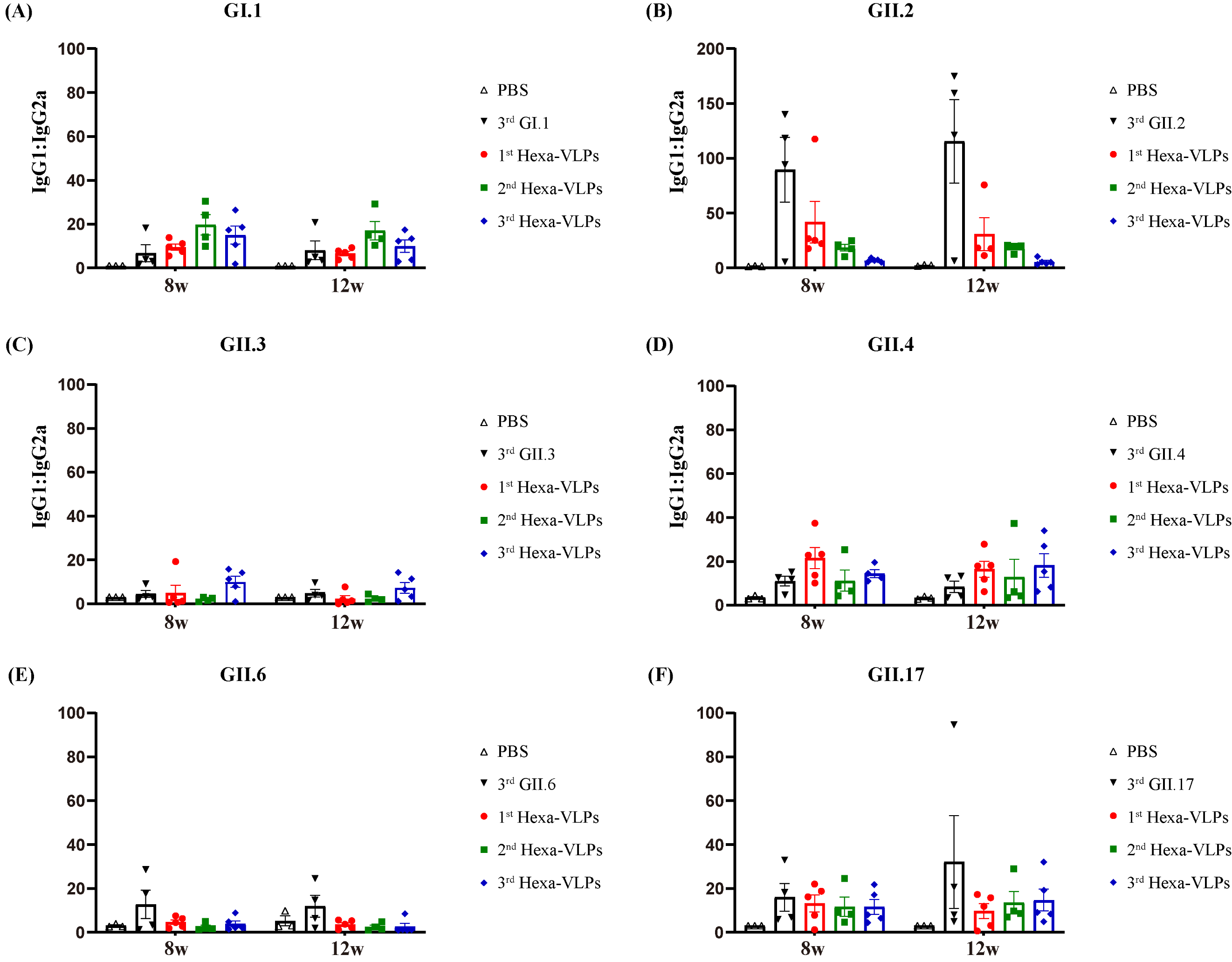


**Supplementary Figure 4.** Calculation of IgG1:IgG2a ratio. IgG1:IgG2a of 8 w sera from different immune regimens against (A) GI.1, (B) GII.2, (C) GII.3, (D) GII.4, (E) GII.6 and (F) GII.17 were calculated and displayed. All data were shown as means ± SEM. Statistical differences between IgG1 and IgG2a of all groups were analyzed using One way ANOVA test with Tukey correction.


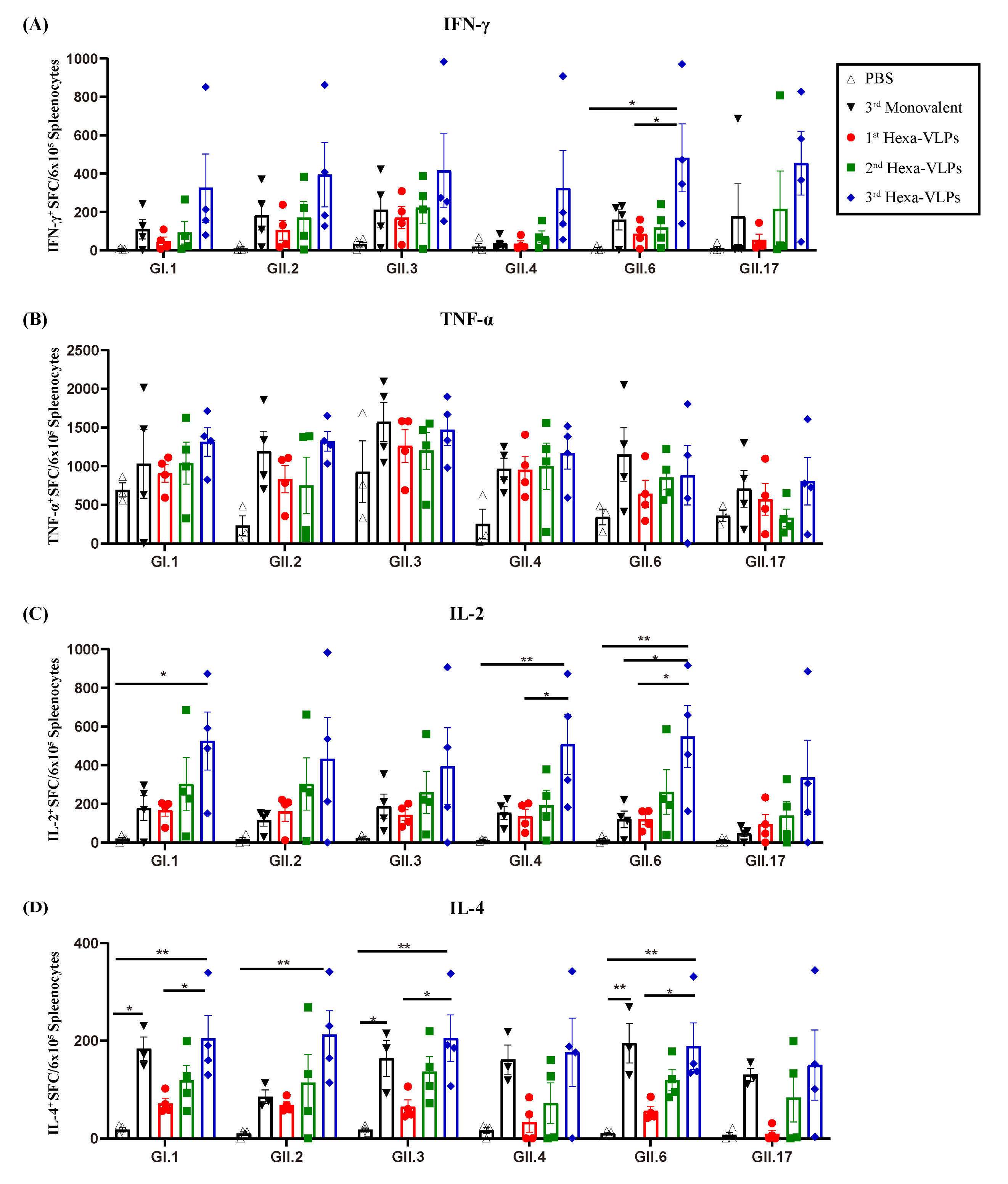


**Supplementary Figure 5.** IFN-γ,TNF-α,IL-2 and IL-4 ELISPOT assays of splenocytes from different immune regimens. BALB/c mice were euthanized at 12 w, splenocytes were restimulated with 10 ug/ml VLPs. (A) IFN-γ, (B) TNF-α, (C) IL-2 and (D) IL-4 positive spots were quantitated using an ELISPOT reader. Results were expressed as the average number of SFC per 6 х 10^5^ input splenocytes. All data were shown as means ± SEM (n=3~5). Statistical differences were analyzed using One way ANOVA test with Tukey correction for multiple testing. p < 0.05 (*) was considered statistically significant; p < 0.01 (**); p < 0.001 (***); p < 0.0001 (****).
